# An enabler or a barrier: implications of local government autonomy for effective primary health care (PHC) reforms in Nigeria

**DOI:** 10.11604/pamj.2026.53.30.49847

**Published:** 2026-01-25

**Authors:** Gafar Bolaji Alawode, Abdul-Rahman Akintomiwa Ajibola, Opemipo Rachael Afolabi, Carlson Nkwain, Kafayat Abiodun Alawode

**Affiliations:** 1 Development Governance International Consult, Federal Capital Territory, Abuja, Nigeria,; 2 United Kingdom Health Security Agency and University of Edinburgh, Edinburgh, United Kingdom

**Keywords:** Local government autonomy, primary health care, Nigeria, decentralization, governance, fiscal federalism

## Abstract

**Introduction:**

in July 2024, Nigeria´s Supreme Court granted autonomy, including financial, to local government, a decision with major implications for primary health care (PHC). Although local governments are constitutionally responsible for PHC, weak fiscal independence and administrative capacity have hindered performance. This study examines opportunities and challenges that LGA autonomy poses for PHC reforms, particularly within the Primary Health Care Under One Roof (PHCUOR) policy.

**Methods:**

a qualitative exploratory policy analysis was conducted using key informant interviews (n=288) and a desk review. Participants were purposively drawn from federal, State, and local levels, including policymakers, LGA officials, civil society, and development partners. Data were analyzed using a hybrid of framework and thematic analysis, guided by study objectives and allowing for emerging insights across governance, financing, accountability, coordination, and service delivery.

**Results:**

stakeholders largely viewed the ruling as a catalyst for grassroots health system strengthening. Nonetheless, concerns were raised about LGA capacity in financial management, service delivery, and alignment with governance structures such as PHCUOR. Key risks include fragmentation of coordination, fiscal mismanagement, and human resource inefficiencies. At the same time, autonomy could enhance responsiveness, accountability, and community engagement if supported with capacity development, oversight, and inclusive governance.

**Conclusion:**

local government autonomy presents both transformative potential and systemic risks for PHC in Nigeria. Realizing its benefits requires institutional capacity building, effective intergovernmental coordination, digital financial systems, and political economy-informed planning. The ruling provides a critical opportunity to recalibrate PHC governance and advance health equity locally.

## Introduction

Primary health care (PHC) is widely acknowledged as the cornerstone of equitable, efficient, and cost-effective health systems, especially in low- and middle-income countries (LMICs) like Nigeria [1]. It plays a crucial role in achieving universal health coverage (UHC) and improving population health outcomes by providing essential services such as immunization, maternal and child health, and health promotion at the community level [2]. Despite its central role in advancing UHC, PHC performance in Nigeria remains weak, reflected in poor health outcomes such as a maternal mortality ratio of 512 per 100,000 live births, neonatal mortality of 41 per 1,000, and under-five mortality rate of 110 per 1,000 live births, among the worst globally [3]. Coverage of essential PHC services, including skilled birth attendance, immunization, and family planning, lags regional and global averages [3,4]. These outcomes are linked to systemic weaknesses in PHC governance. While the primary responsibility for PHC delivery is on the local government, [5] the tier is constrained by fragmented authority, weak accountability, and heavy dependence on State governments for finance and administration [5,6]. The joint State-LGA account system further entrenched political interference, restricting fiscal space and decision-making at the local government level [5,6]. This misalignment between PHC responsibilities and resources has perpetuated inefficiencies, service gaps, and inequities in access [5]. In response, the government launched the Primary Health Care Under One Roof (PHCUOR) policy in 2011, consolidating leadership, resources, and service delivery under State primary health care development agencies/boards [7]. Primary health care under one roof has contributed to improvements in policy alignment, integration of vertical programmes, and community participation through ward development committees. However, its implementation remains uneven, and its effectiveness has been hampered by persistent LGA dependence on State governments and the absence of genuine fiscal autonomy [5].

Nigeria has a long history of attempts at reforming these local government-level concerns, shaped by broader political struggles over federalism and grassroots governance. Reforms since the 1976 local government reform have focused on four recurring themes: i) entrenching democratic governance at the local level; ii) securing financial autonomy; iii) improving accountability and efficiency, and iv) reducing political interference by State governments [8-11]. Yet, these efforts have repeatedly stalled due to State-level dominance and weak institutional capacity at the LGA level. In July 2024, Nigeria´s Supreme Court ruled in favour of granting autonomy to LGAs, declaring State-appointed caretaker committees unconstitutional and allowing the direct disbursement of LGA allocation from the federation account to the LGA account [12]. This ruling provides a potential opportunity to correct systemic governance distortions and enhance the performance of PHC at the grassroots. However, it also introduces significant risks, including capacity constraints at the local government level, weakened coordination with State-level PHC structures, and risks of financial mismanagement in the absence of robust accountability systems [13]. The evidence on health sector decentralization from other countries offers both cautionary tales and useful lessons. In contexts like Ghana, Zambia, Uganda and the Philippines, decentralization has enabled localized health planning and budgeting but also exposed gaps in coordination and accountability where institutional capacity is weak [14]. In Nigeria, studies have highlighted the dysfunctional nature of LGA administration and its impact on PHC service delivery [13,15]. Yet, to date, little empirical work has systematically examined the likely implications, both positive and negative, of implementing LGA autonomy within Nigeria´s specific federal and health governance landscape. This study responds to that gap by exploring the implications of local government autonomy for PHC reform in Nigeria. Specifically, the study aims to i) enhance stakeholder understanding of the spirit and letter of the Supreme Court judgement on LG autonomy, including its legal, operational, and governance implications for PHC management; ii) appraise the implications of LG autonomy for PHC management generally and specific health sector policy thrusts, such as PHCUOR; iii) analyze the roles, influence, and interests of actors and institutions in shaping outcomes of autonomy, and their impact on PHC governance; iv) identify strategies for mitigating potential risks and leveraging opportunities from LG autonomy, focusing on actionable, context-specific recommendations for PHC improvement in Nigeria.

## Methods

**Study design:** this descriptive and exploratory research adopted a qualitative research design, comprising key informant interviews and document reviews. Semi-structured questionnaires were used to gather insights from stakeholders with varied roles, interests and influence within the health financing and governance space at the federal, State and local government levels, including policymakers, State and LGA-level implementers, technical advisors, LGA authorities, civil society organizations, etc. These interviews complemented document reviews by providing contextual background and aiding data triangulation for enhanced validity and reliability. A COREQ-aligned reporting framework was applied across the research process, ensuring transparency in researcher characteristics, participant selection, data collection, and analytical procedures.

**Research team and reflexivity:** the research team consisted of five investigators (3 males and 2 females), three based in Nigeria and two in the United Kingdom, most with academic and professional backgrounds in public health, health financing, governance, and policy research. Data collection was carried out by twelve trained research assistants, one assigned to each study State, who had prior experience in qualitative interviewing and stakeholder engagement. These research assistants were supervised by the study authors, all of whom have extensive experience conducting qualitative research within the Nigerian health system. There were no pre-existing personal relationships between the interviewers and the participants. Before each interview, participants were informed about the purpose of the study, the roles and institutional affiliations of the research team, and how the data would be used. Throughout the study, the team remained attentive to issues of positionality and potential bias. Given their prior involvement in health financing and governance work in Nigeria, the researchers maintained reflexive notes and engaged in regular debriefing sessions to ensure neutrality, enhance self-awareness, and minimise the influence of assumptions on data collection and analysis.

**Study setting:** the study was conducted in Nigeria, a federal republic composed of 36 States and the Federal Capital Territory, grouped into six geopolitical zones. Nigeria operates a three-tier governance structure: federal, State, and local governments [16]. To ensure national representativeness and capture variations in PHC situations across the country, two States were randomly selected from each of the six geopolitical zones, resulting in a total of 12 States included in the study. In each selected State, one local government area was randomly chosen from each senatorial district, resulting in three local government areas per State and a total of 36 local government areas involved in the study, as shown in Table 1. This approach aimed to provide a holistic view of local government autonomy and its implications for diverse governance and health system structures.

**Table 1:** geopolitical zones in Nigeria, and the 12 selected States and 36 local government autonomy included in the study

S/N	Geopolitical zones	Selected states	Selected local government areas
1	South-West	Oyo	Ibadan North, Ogbomoso South, and Akinyele
		Osun	Olorunda, Obokun and Ife North
2	South-East	Ebonyi	Ikwo, Ezza South, and Afikpo South
		Anambra	Awka South, Onitsha South, and Orumba North
3	South-South	Rivers	Port Harcourt City, Oyigbo and Ahoada West
		Cross-River	Calabar Municipal, Ogoja and Obubra
4	North-Central	Nasarawa	Keffi, Akwanga and Lafia
		Kwara	Offa, Ilorin West and Moro
5	North-West	Kaduna	Sabon Gari, Makarfi, and Giwa
		Sokoto	Sokoto North, Gwadabawa, and Dange Shun
6	North-East	Adamawa	Yola North, Mayo Belwa, and Mubi North
		Bauchi	Misau, Diade and Bauchi

**Study population and sampling strategy:** the study employed purposive sampling to select 288 key informants at the federal, State, and local levels, with no repeat interviews. At the federal level, participants were drawn from institutions such as the Federal Ministry of Health and Social Welfare, Association of Local Governments of Nigeria (ALGON) headquarters, development partners, and civil society organizations (CSOs). State-level respondents were selected from the State ministries of health, State primary health care development agencies/boards (SPHCDAs/SPHCBs), ministries of local government, local government service commissions, ministries of finance, development partners and CSOs. Local government-level informants included LGA Chairpersons or their deputies, LGA Secretaries, heads of department of the health, and National Union of Local Government Employees (NULGE) representatives. Stakeholders were selected based on their knowledge, experience, interest, and influence over local government affairs, PHC management, accountability, and the implementation of PHC policy thrusts. The sampling strategy ensured inclusion of actors with formal authority, operational roles, and external oversight functions. Non-participation, mostly because of unavailability, was recorded on a few occasions [6], and replacements were made from similar cadres to maintain representativeness.

**Data collection:** semi-structured interview guides were developed for each stakeholder group and tailored to their specific roles in governance, PHC management, and accountability. Key informant interviews were conducted to collect qualitative data from the 288 selected participants. Appointments were scheduled through formal letters and emails. Interviews were conducted both in-person and virtually, depending on feasibility, and each session lasted between 30 and 60 minutes, without a third party, and was recorded with participants´ consent. Field notes were also taken to supplement audio recordings. Transcripts were not returned to participants for correction; however, clarifications were sought during interviews when necessary. In addition, document review was conducted to provide further understanding and validate KII findings against existing literature, reports, policies, and legal frameworks, thereby ensuring more comprehensive and triangulated insights. The review followed the READ approach: i) ready materials; ii) extract data; iii) analyse data and iv) distil findings [17]. The acquisition of documents was facilitated through stakeholder interviews, during which relevant materials were requested and obtained.

**Data analysis and synthesis:** a team of four analysts independently reviewed the transcripts using NVivo. Qualitative data were analysed using a hybrid approach that combined framework analysis, a systematic and rigorous method developed by Ritchie and Spencer for applied policy research [18] and thematic analysis. While framework analysis provided a structured, objective-driven lens, the thematic analysis allowed for the emergence of new insights beyond the predefined codes. The integration of these methods together enabled the identification of both predefined and emerging themes, which are presented as the study´s findings. The framework analysis was selected due to its distinctive matrix-based structure that facilitates case-by-case and thematic analysis while maintaining clear connections between raw data and emergent findings [18]. The resulting framework, the data extraction matrix, had rows representing key informants, columns representing codes (informed by the research objectives), and cells containing synthesized summaries of responses per theme. This structure enabled transparent data management and provided a clear audit trail that enhanced the credibility of the analytical process [18]. A validation meeting was held with stakeholders to provide input and further feedback on the initial data collected through KIIs and desk review.

**Ethical considerations:** this study did not require formal ethical approval, as it did not involve the collection of sensitive or personally identifiable information from respondents. The research focused on governance structures, health systems, and institutional arrangements related to PHC delivery and local government administration in Nigeria and no individual-level data or private information were obtained. The research adhered to principles of integrity, transparency, and respect for participants.

## Results

**Summary of the Supreme Court judgement on local government autonomy:** the Supreme Court´s landmark judgement on local government autonomy in Nigeria addressed two key areas: democratic governance and financial autonomy (Table 2). It affirmed that LGAs must function as elected councils, rendering caretaker committees unconstitutional, and ruled that LGAs are entitled to direct allocations from the federation account, limiting State interference and reinforcing fiscal independence. While the ruling did not explicitly reference health, its implementation has significant implications for PHC governance and financing, with potential ripple effects on the wider health system.

**Table 2:** summary of the Supreme Court judgement on local government autonomy in Nigeria

S/N	Themes	Descriptions
1	Democratic governance at the LG level	Declarations 1, 2, 3, 4, 5, and 8 emphasized that state governments cannot lawfully dissolve democratically elected local government councils or replace them with caretaker committees. They affirm that: i) only democratically elected LGCs are constitutionally recognized under Section 7(1) of the 1999 Constitution; ii) any dissolution by state governors or assemblies through laws, executive orders, or other means is unconstitutional, null, and void; iii) Declaration 8 specifically states that such actions amount to gross misconduct by state officials
2	Financial autonomy & direct allocation from the federation account	Declarations 6, 7, 9, 10, 11, 12, 13, 14, and 15 focus on ensuring that local government funds from the federation account are paid directly to them, bypassing state governments. They affirm that: i) State governments have no constitutional right to control, manage, or disburse LGC allocations; ii) Section 162 (3) & (5) of the 1999 constitution allows direct payment to LGCs; iii) The current practice of states holding LGC funds is unconstitutional iv) Orders 13, 14, 15 mandate immediate compliance, requiring the federal government to directly pay LGCs and restraining states from further interference

LGA: local government authority; LGC: local government councils

**Thematic insights on local government autonomy and primary health care delivery:** this section presents study insights as eight themes derived from triangulated evidence from key informant interviews and desk review. The themes reflect perceptions of the Supreme Court ruling on LGA autonomy, current LGA governance, potential impacts on governance and PHC delivery, as well as anticipated challenges and unintended consequences.

*Theme 1: stakeholders´ understanding of the Supreme Court ruling:* this theme examines the different interpretations and expectations of stakeholders concerning the recent Supreme Court ruling on local government autonomy. Opinions varied from hopeful expectations of enhanced grassroots governance to cautious concerns about implementation challenges and capacity limitations.

**Positive perceptions of the Supreme Court ruling:** for many stakeholders, the autonomy ruling is a welcome development. The Chairman of the Nigeria Governors´ Forum, Kwara State Governor, AbdulRazaq AbdulRahman, expressed relief, noting that the decision alleviates the financial burden States have historically shouldered [19]. Similarly, ALGON commended the judgement as a victory for local governance, with its national president, Aminu Muazu-Maifata, asserting that the ruling would enhance the financial independence of the 774 local government councils in Nigeria [20]. In the health sector, Professor Ali Pate, the Coordinating Minister of Health and Social Welfare of Nigeria, described the ruling as a critical milestone in health care reform [21].

**Concerns about the implementation of the Supreme Court ruling:** despite optimism, some stakeholders doubted State actors´ political will to allow full autonomy, warning that implementation could face jurisdictional contestations. Others noted uneven understanding and interpretation across levels, with some cases potentially requiring judicial clarification. As one respondent observed: *"Despite the Supreme Court judgement, there will be a call for interpretation... these are the other windows that are open for subsequent discussion”*. Additionally, several State-level stakeholders raised concerns about LGAs' limited capacity to manage finances effectively. The risks identified include limited institutional capacity, weak accountability mechanisms, and potential corruption, which could undermine PHC delivery. The removal of State Joint Government Account (SJLGA); oversight role heightens fears of mismanagement.

**Misconceptions about the Supreme Court ruling:** there is a common misconception among stakeholders that the Supreme Court ruling on local government autonomy contradicts Section 162 (6) of the 1999 Constitution [16]. However, a closer examination of the ruling reveals that no contradiction exists. The Supreme Court did not entirely prohibit allocations through the SJLGA; rather, it clarified that the Federal Government has the discretion to either pay funds directly to LGAs or channel them through the SJLGA [12]. This interpretation highlights that while the Supreme Court ruling provides an avenue for direct LGA funding, it does not outrightly abolish the SJLGA. However, some stakeholders argue that amending the Constitution to explicitly eliminate the SJLGA would provide greater clarity and reinforce fiscal autonomy for LGAs.

*Theme 2: alignment between the ruling and existing policy:* the PHCUOR policy aligns with the National Health Act (2014), which emphasizes coordination and mandates SPHCDAs to oversee PHC services [22]. In practice, SPHCDAs are funded mainly through deductions from the SJLGA, managed by the State government, as stipulated in many State laws. However, the local government autonomy ruling has created a misalignment with existing policies and legal frameworks governing PHC delivery, like the PHCUOR policy, which relies heavily on the current system of fund remittances from LGAs to SPHCDAs [23]. In response to the Supreme Court ruling, some States have begun to introduce new legal frameworks that may undermine the intended autonomy of LGAs. For example, Anambra State has introduced the Local Government Administration Law, 2024, which establishes a Local Government Consolidated Account and a Local Government Joint Security Trust Account [24]. This law mandates LGAs to remit a percentage of their allocations to these accounts, effectively reintroducing State control over LGA funds. This move directly contradicts the spirit of the Supreme Court ruling and could undermine LGA autonomy, particularly in funding PHC.

*Theme 3: institutional coordination:* the PHCUOR policy was introduced to address the fragmentation in PHC governance and service delivery in Nigeria. By unifying responsibilities under the State Primary Health Care Development Agencies, the policy aimed to streamline governance, reduce duplication, and improve efficiency [7]. However, with LGA autonomy, concerns have been raised about the potential erosion of these coordination gains.

**Centralized fund pooling:** pooling LGA funds under SPHCDAs has been central to improved planning, procurement, staff development, and PHC coordination. This pooling of funds allowed for more centralized financial planning, resource allocation, and service delivery coordination [23]. It also facilitated investments in infrastructure, procurement of essential medicines, staff training, and health campaigns, thereby improving overall PHC service quality and equity across LGAs. Some key positive effects of centralized fund pooling include: i) Improved health care services: while full implementation of PHCUOR has not been achieved in all areas, States that have implemented the policy show significant improvements in health care coordination and service delivery. This is particularly evident in the increased availability of essential medicines, enhanced PHC funding, and facility upgrades [23]; ii) enhanced coordination and efficiency: findings revealed that PHCUOR has led to clearer roles between the State and LGA health institutions which has led to better health care delivery, reduced duplication of efforts, and more efficient use of resources by bringing different services and health care programs together. This makes it easier to implement comprehensive health strategies, such as maternal and child health programs, chronic disease management, routine immunization, etc. [23,25].

*Theme 4: operational and administrative capacity:* capacity limitations across institutional arrangements, human resource management, and financial management emerged as a major barrier to the successful implementation of LGA autonomy [25,26]. Stakeholders emphasized that without deliberate investments in training, mentorship, and systems strengthening, local governments may lack the tools to manage autonomy effectively, particularly for PHC delivery. The identified capacity gaps are highlighted below.

**Governance and institutional weaknesses:** the findings reveal that many LGAs lack foundational governance structures and administrative systems needed to operationalize autonomy effectively. Weak oversight, inadequate leadership training, and poor intergovernmental coordination limit the ability to make informed decisions and manage resources effectively. Stakeholders stressed the need to modernize operations through digitization and systemic reforms, as emphasized by a respondent: *"They need to move from analogue to digital. They need to digitize their activities and strengthen coordination at that level."*

**Human resource constraints:** studies have consistently reported a shortage of skilled personnel within LGAs, particularly in financial management, health workforce planning, and administrative roles. High turnover rates, lack of incentives, and inadequate training opportunities exacerbate these shortages [27]. Many LGAs struggle to retain qualified personnel due to poor remuneration and career advancement opportunities. Without skilled personnel, effective governance and service delivery remain elusive [27].

**Financial management deficiencies:** the study uncovered significant challenges of budgeting and financial planning at the LGA level. Many LGAs lack the technical expertise to develop and implement realistic budgets, track expenditures, and ensure accountability in financial resource allocation [28]. This gap often results in mismanagement of funds, delayed salary payments, and inefficiencies in PHC service provision.

**Service delivery and infrastructure deficiencies:** it was reported that many health facilities at the LGA level lack essential medical equipment, adequate infrastructure, and sufficient health care personnel to deliver PHC services to meet the needs of their populations. Poor supply chain management systems result in frequent stockouts, while limited managerial capacity affects the quality of services delivered [4].

*Theme 5: service delivery:* service delivery is a core function of primary health care and a critical determinant of health outcomes at the community level. Persistent issues such as human resource shortages, inadequate infrastructure, poor drug supply chains, and limited financial protection mechanisms (e.g., health insurance) continue to undermine the effectiveness of PHC services [4]. In the context of local government autonomy, the capacity of LGAs to deliver equitable, efficient, and quality health services has come under increased scrutiny as highlighted below.

**Coverage of essential services:** disparities exist in the geographical coverage of PHC services in Nigeria, with rural communities being mostly underserved. Similar trends have been observed in other LMICs, where rural areas often experience limited access to PHC services, a shortage of health care providers, and generally poorer health outcomes compared to urban populations [29,30]. While many States have made progress, such as renovating PHC facilities and expanding service coverage, significant challenges persist, particularly in the equitable distribution of health workers, consistent supply of essential commodities, and routine maintenance of infrastructure etc. [4].

**Quality of services:** stakeholders opine that LGAs´ dependency on the State government has limited their ability to independently deliver essential services; thus, increased financial and administrative autonomy could enhance service delivery efficiency and responsiveness. *“Due to the current arrangement, we depend on the State government for the delivering of essential services in health care, water sanitation, hygiene and education. But autonomy could help us do more with availability of resources in the LGAs.”*

*Theme 6: financial accountability:* financial accountability in Nigeria´s Local Government Authorities varies significantly due to differences in governance structures, historical financial practices, and political dynamics.

**Governance structures and financial accountability mechanisms:** the study found that some governance structures are in place to ensure financial accountability, though their implementation remains inconsistent [28]. The SJLGA previously granted State governments control over LGA finances, resulting in delays, fund diversions, and a decline in LGAs´ fiscal independence. *“The joint account system, established by law, governs the flow of funds between States and LGAs. This system, housed within the Ministry of Local Government, ensures that States manage and disburse funds to LGAs. While intended for accountability, this setup restricts LGAs´ direct access to their funds*.” The Office of the Auditor-General for Local Governments provides oversight but is constrained by political interference and weak enforcement. Audits are often irregular or ignored, while public accounts committees remain inactive, and financial records are rarely reviewed. These gaps, compounded by limited independence and State interference, undermine accountability and heighten risks of mismanagement and inefficient service delivery.

**Transparency and financial tracking mechanisms:** a significant challenge in LGA financial management is the absence of transparency in fund allocation. Local government areas’ budget information is not publicly disclosed, which hinders effective monitoring [28]. Also, local communities are often excluded from financial decision-making, resulting in misaligned spending priorities. *“People just spend the money, and you don't even know how that money was spent.”*

*Theme 7: political economy analysis of local government autonomy financial:* the political economy of local government autonomy in Nigeria reflects the interplay of power, interests, and institutions across federal, State, and local levels. Although the Supreme Court ruling seeks to strengthen grassroots governance and PHC, its implementation is shaped by political incentives, State control, and concerns over local accountability and capacity. Understanding these dynamics is key to identifying factors that enable or constrain the realization of true autonomy.

**Key actors and institutions whose influence affects the implementation of local government authority:** this policy analysis has delineated five distinct categories of stakeholders whose influence is pivotal in the execution of LGA autonomy. Table 3 shows the key actors and institutions that influence the implementation of LGA autonomy. It categorizes stakeholders in line with the multi-layered governance system from federal executives down to community participants.

**Table 3:** key actors and institutions whose influence affects the implementation of local government autonomy in Nigeria

Category	Actor/Institution	Description/Role	Interest	Influence on LGA
**Federal-Level Actors**	Presidency	Oversees national policy implementation	To ensure policy alignment and national health goals are achieved; To ensure the constitution of Nigeria is rightly interpreted; to uphold standards and equity	They influence policy direction and funding structures that LGAs must comply with
	Health MDAs	Formulates national health policies and guidelines		
	Association of local governments of Nigeria (ALGON)	Advocates for local government autonomy and represent LGA interests		
	Federal account allocation committee	Distributes financial resources to the three tiers of government		
	Attorney general of the federation (AGF)	Initiated the legal suit that enforced the constitutional autonomy of the 774 local government areas		
**State-level actors**	Governor	Oversees state policy implementation and governance	To retain control over local government authority resources and service delivery; To maintain political and administrative oversight	They often dominate LGA decision-making and funding through political and financial control
	State houses of assembly	Formulate state laws and exercise local government authority oversight		
	State ministries of health	Formulate health policy and provide public health services		
	State ministries of Local government affairs	Oversee state-local government authority relationships convene and oversee LGA fund disbursement through JAAC		
	State primary health care boards	Implement PHCUOR policy to manage primary health care services statewide		
	Auditor general’s office of LGAs	Audit LGA financial statements and operations for accountability and transparency		
	State Ministries of budgets and economic planning	Prepare and analyze state budget and economic development strategies		
	LGA service commission	Serves as the key human resource management body within the local government authority system		
**Local government level actors**	LGA chairmen	Oversees local government authority administration and governance	To exercise autonomy and improve local service delivery; To gain financial control to meet constituents’ expectations	They are constitutionally empowered but often limited by state-level dominance and weak capacity
	Directors of primary health care	Manage and oversee primary health care services within the LGA		
	Heads of local government authority departments	Lead various local government authority departments for effective service delivery		
	Statutory committees	Facilitate PHC implementation and coordination at local government authority level		
**Community-level actors**	Traditional leaders	Facilitate grassroots engagement and representation	Improved access to quality health services; Participatory planning and implementation	They have strong local knowledge and legitimacy, but are often underutilized
	Youth leaders	Serve as representatives of youth advocating for their interests and needs		
	Women leaders	Serve as representatives of women's interests and needs		
	Chairs of community development committees	Lead the implementation of local development initiatives		
	Community participants	Benefit from governmental policies and programs		
**Development partners and civil society groups**	Development partners	External organizations supporting development initiatives	To promote sustainable development and equitable service delivery; To strengthen local governance systems	They provide crucial support roles (technical and financial)
	Civil Society Groups	Non-governmental organizations advocating for community interests		
	Professional associations and unions	Professional bodies representing sector-specific interests		

LGA: local government authority; JAAC: joint accounts allocation committee; MDAs: ministries, departments and agencies; PHCUOR: primary health care under one roof; PHC: primary health care

*Theme 8: unintended consequences of local government autonomy:* despite the intended benefits of the Supreme Court ruling, it has introduced several potential unintended consequences that could impact governance, including PHC service delivery. This section reflects the findings of this study on these unintended effects.

**Fragmentation in primary health care coordination:** while local government autonomy aims to improve responsiveness and accountability, stakeholders cautioned that it could fragment the PHC system. Under PHCUOR, pooled funding and centralized oversight enabled coherent planning, resource use, and accountability [23]. Autonomy, however, allows each LGA to manage resources independently, which may erode these gains. Without State-led coordination, stakeholders warned of inefficiencies, duplication, and diminished PHC impact.

**Financial mismanagement by local governments:** local government autonomy was intended to give councils greater control over resources for local development. However, past experiences and stakeholder concerns highlight persistent risks of mismanagement. During earlier periods of autonomy, corruption and misuse of funds were common, leading to inefficiencies. As one stakeholder noted: *“When this democracy started, LGAs had more autonomy… some chairmen abused it, spent recklessly, and there was nothing to show for it*.”

**Human resource mismanagement:** before autonomy, LGAs faced major human resource challenges, including overemployment, poor recruitment, and underemployment [31]. Stakeholders warned that autonomy could worsen these practices if oversight and accountability remain weak, inflating wage bills and diverting resources from critical sectors like health. Without safeguards, autonomy may reinforce rather than resolve HRM problems.

**Inequitable revenue distribution:** before the implementation of financial autonomy, funds allocated to local governments were pooled into the SJLGA and distributed equitably among LGAs, ensuring that both urban and rural areas received financial support regardless of their revenue-generating capacities [6]. Despite its flaws, this system provided a form of financial balance, preventing extreme disparities in resource allocation, especially for rural local government areas that lack significant internally generated revenue (IGR). Now, with autonomy, LGAs will receive their funds directly [12]. While this gives them more control, it may also lead to inequality. Wealthier LGAs that can generate more income may thrive, while poorer or rural ones struggle to provide basic services due to limited resources. If it is not carefully managed, autonomy could unintentionally widen the gap in service delivery across regions.

**Political independence and accountability:** before autonomy, local government chairmen operated under the strong influence of State governors, who often handpicked them to align with their political agendas [6]. This arrangement meant that LGAs had limited independence in decision-making, as financial and administrative control largely rested with the State government. While this centralized oversight sometimes ensured coordination in resource allocation, it also restricted local governments from independently managing their affairs, making them extensions of the State´s political structure rather than autonomous governance entities [32]. However, with autonomy, chairmen and LGA officials will have greater control over local decision-making. A positive consequence of this shift is that it may gradually reduce overreliance on State-level directives and promote stronger political accountability at the local level.

## Discussion

The Supreme Court ruling for local government autonomy financial autonomy alters PHC governance in Nigeria, empowering LGAs to determine and own health spending priorities. This shift in governance structures at the local government level is supported by perspectives from academia and policymakers who assert that when local governments have decision-making authority and fiscal independence, they can be more responsive to community health needs and priorities [33]. It has also been argued that such reforms are more complex and diverse than conventionally accepted [34]. For LGAs to achieve effective and sustainable decentralization as mandated by the Supreme Court ruling, strategic health initiatives aimed at strengthening the health system and improving access to health services need to be tailored to the specific needs of each local government area. However, while this could potentially strengthen grassroots democratic health system governance based on local health needs instead of State directives, it also challenges the principles of PHCUOR reform, which hinges on “One management, one plan, one monitoring and evaluation systems” at the State level [35]. Without strong health coordination mechanisms, there is a risk that PHC programs across the LGAs will become disjointed, as was the case in Kenya where devolution resulted in disruption, interference and confusion in the management of human resources for health, as well as significant delays in procurement and prolonged stock-outs of essential medicine and medical supplies in health facilities [36].

In the context of financial management and accountability, financial autonomy for LGAs entails the need for financial responsibility. However, stakeholders have strong concerns about the LGAs' capacity to effectively manage the increased financial resources. Without adequate and robust financial transparency and accountability mechanisms in place, there is a significant risk of recycling the existing mismanagement of funds and politically motivated spending experienced with the PHCUOR reforms prior to the Supreme Court ruling [37]. Fiscal management and the resulting misallocation of funds and inefficient spending are well-documented in Nigeria, a trend similar to that in Uganda, which has exacerbated inefficiencies under decentralization [37,38]. However, the Supreme Court ruling presents a window of opportunity to put in place or strengthen financial mechanisms to mitigate fiscal mismanagement, including downward accountability to communities, as reported in Turkey [39] or performance-based financing to strengthen accountability as reported in Rwanda and Zambia [40,41]. The political economy landscape at the local government level in Nigeria is characterized by long-standing political patronage dynamics among competing interests and power struggles among various stakeholders. The Supreme Court ruling on LGA financial autonomy threatens the political interests of those seeking to maintain patronage networks and influence at the grassroots level. This tension and competing interests could potentially hinder the effective implementation of health reforms under the new framework of health financing and governance in Nigeria [42]. In addition, the ruling elevates the role of LGAs as critical actors in health governance, with growing influence on the implementation of health policy thrusts such as the sector-wide approach (SWAp), and other State and national health priorities. To maximize these opportunities, there is a pressing need to institutionalize mechanisms for engaging LGAs not only in service delivery but also in the co-design, implementation, and accountability processes of health policies. Without such deliberate engagement, LGAs risk remaining passive implementers rather than active contributors to policy coherence and health system strengthening. The perceived primary benefit of the Supreme Court ruling for PHC in Nigeria is to enhance access to and delivery of health services by increasing responsiveness and agility to local needs and priorities.

**Strengths and weaknesses of the study:** this study had notable strengths, including wide geographic coverage across all six geopolitical zones and a large, diverse sample of key informants from federal, State, and local government levels, enhancing the generalizability of findings. Methodological triangulation of key informant interviews and document review enhanced the robustness and credibility of findings, and the use of a systematic framework analysis also ensured transparency and clear links to the original data. However, as a qualitative inquiry, the findings are context-dependent and not statistically generalizable. Respondent perspectives may also reflect institutional or political biases, while access to relevant documents varied across States. Finally, the study captures views at an early stage of the Supreme Court ruling´s implementation, and evolving dynamics may lead to shifts not reflected in this analysis.

**Study implications/recommendations:** the Supreme Court´s ruling on local government autonomy in Nigeria presents both opportunities and challenges for the delivery of PHC. To maximize the potential benefits while mitigating associated risks, the following recommendations are proposed, based on the study´s findings:

**Strengthen State-local government authority coordination:** effective governance of PHC under the new autonomy framework requires stronger mechanisms for intergovernmental collaboration. This includes establishing a functional State-LGA PHC Forum to facilitate continuous dialogue between State and LGA stakeholders, and revitalizing State Councils on Health to ensure regular and meaningful engagement.

**Promote financial accountability at the local level:** to enhance fiscal responsibility, local governments should adopt digitized financial management systems and establish citizen-led resource tracking mechanisms. Greater community participation in health governance should be promoted, alongside peer-comparison platforms among local government areas to encourage accountability and performance improvement.

**Build local capacity for primary health care management:** strengthening the technical and managerial capacity of LGAs is fundamental for effective service delivery. This requires targeted investments in health leadership development, the operationalization of a clearly defined minimum service package at the local government level, and provision of structured technical and leadership training programs.

**Harmonize the legal and policy environment:** a coherent legal and policy framework is critical for sustainable PHC delivery. This involves reviewing and amending conflicting laws while building consensus on sustainable PHC financing. Clearly delineating the financial commitments of federal, State, and local governments will also be essential to avoid duplication and resource gaps.

**Promote health investment at the local government authority level:** local government authority should be mandated to prepare and adequately fund annual health operational plans that are aligned with performance targets and State-level priorities. Strengthening resource-tracking mechanisms and introducing performance-based incentives for LGAs will help drive results. Targeted investment should be prioritized in maternal, newborn, and child health (MNCH), health insurance, and health security.

**Incorporate political economy analysis into primary health care planning:** an agile management approach to PHC delivery requires embedding political economy analysis within planning and implementation processes. This ensures that reforms are not only technically sound but also politically and institutionally feasible. Key actions include conducting regular political economy appraisals and strategically engaging political, traditional, and community stakeholders to build broad-based support.

**Align with ongoing reform initiatives:** to ensure coherence and sustainability, LGA reforms should be aligned with broader national initiatives. This includes leveraging the political influence of the ALGON to advance PHC revitalization, aligning efforts with flagship programs such as the Maternal and Neonatal Mortality Reduction Innovation Initiative (MAMII), and embedding local government autonomy reforms within the SWAp to harmonize donor and government investments.

**Meaning of the study:** this study highlights that the Supreme Court ruling on local government autonomy marks a pivotal opportunity to reshape PHC governance in Nigeria. Beyond the legal framework, it underscores the need to translate fiscal independence into stronger local leadership, improved accountability, and more responsive service delivery. The findings suggest that realizing the potential of this reform depends on aligning political incentives, building institutional capacity, and ensuring that autonomy leads to measurable health outcomes rather than administrative fragmentation.

**Unanswered questions and future research:** key questions remain on how the implementation of LGA autonomy will affect the quality, equity, and sustainability of PHC delivery across diverse State contexts. Further research is needed to assess how States and LGAs adapt to their new roles, what accountability mechanisms are most effective, and how community participation can be institutionalized in health governance. Longitudinal studies examining the impact of these governance shifts on health outcomes will be critical for guiding future policy and reform implementation.

**Limitations:** this study has a few limitations that may influence the interpretation of its findings. The purposive sampling approach may have introduced selection bias, though this was minimised through broad stakeholder mapping. Political sensitivities could also have led to social desirability bias, which was mitigated through probing during interviews. A stakeholder validation meeting further strengthened the credibility of findings. Nonetheless, results should be interpreted with caution regarding their generalizability and applicability to broader contexts.

## Conclusion

The July 2024 Supreme Court ruling on local government autonomy represents a pivotal moment for Nigeria's primary health care system. This policy analysis reveals both the transformative potential and implementation challenges of this landmark decision. While stakeholders perceive the ruling as essential for democratic governance and grassroots health system strengthening, significant concerns persist regarding LGA capacity for financial management, institutional coordination, and service delivery. The study findings show critical tensions between autonomy and coordination. Financial independence empowers LGAs to respond to local health priorities yet threatens the integrated approach of the PHCUOR policy that aimed to reduce fragmentation. Historical evidence of fiscal mismanagement at local levels, coupled with weak accountability mechanisms and political patronage dynamics, raises legitimate concerns about whether autonomy will improve or compromise PHC delivery.

### What is known about this topic

- Fiscal and administrative centralization has historically limited the effectiveness of local governments in managing and financing primary health care services in Nigeria;
- Political economy factors such as State-level control over local government authority finances and weak accountability structures have constrained the realization of true local governance in the health sector.

### What this study adds

- Our study provides empirical insights into how the Supreme Court ruling on local government autonomy could reshape the political and institutional landscape for primary health care delivery in Nigeria;
- Based on our findings, this study offers actionable recommendations to build PHC system resilience through improved State-LGA coordination, financial accountability, and politically informed program management in the context of local government autonomy.
